# Exploring the contribution of efflux on the resistance to fluoroquinolones in clinical isolates of *Staphylococcus aureus*

**DOI:** 10.1186/1471-2180-11-241

**Published:** 2011-10-27

**Authors:** Sofia Santos Costa, Celeste Falcão, Miguel Viveiros, Diana Machado, Marta Martins, José Melo-Cristino, Leonard Amaral, Isabel Couto

**Affiliations:** 1 Grupo de Micobactérias, Unidade de Microbiologia Médica, Instituto de Higiene e Medicina Tropical, Universidade Nova de Lisboa (IHMT, UNL), Rua da Junqueira, 100, 1349-008 Lisbon, Portugal; 2 Centro de Recursos Microbiológicos (CREM), Faculdade de Ciências e Tecnologia, Universidade Nova de Lisboa, Quinta da Torre, 2829-516 Caparica, Portugal; 3 COST ACTION BM0701 (ATENS; 4 Unidade de Parasitologia e Microbiologia Médica (UPMM), Instituto de Higiene e Medicina Tropical, Universidade Nova de Lisboa; Rua da Junqueira, 100, 1349-008 Lisbon, Portugal; 5 UCD School of Public Health, Physiotherapy and Population Science, UCD Centre for Food Safety, Veterinary Sciences Centre, University College Dublin, Belfield Dublin 4, Ireland; 6 Centro Hospitalar Lisboa Norte E.P.E., Instituto de Microbiologia, Instituto de Medicina Molecular, Faculdade de Medicina, Universidade de Lisboa, Avenida Professor Egas Moniz, 1649-028 Lisbon, Portugal

## Abstract

**Background:**

Antimicrobial resistance mediated by efflux systems is still poorly characterized in *Staphylococcus aureus*, despite the description of several efflux pumps (EPs) for this bacterium. In this work we used several methodologies to characterize the efflux activity of 52 *S. aureus *isolates resistant to ciprofloxacin collected in a hospital in Lisbon, Portugal, in order to understand the role played by these systems in the resistance to fluoroquinolones.

**Results:**

Augmented efflux activity was detected in 12 out of 52 isolates and correlated with increased resistance to fluoroquinolones. Addition of efflux inhibitors did not result in the full reversion of the fluoroquinolone resistance phenotype, yet it implied a significant decrease in the resistance levels, regardless of the type(s) of mutation(s) found in the quinolone-resistance determining region of *grlA *and *gyrA *genes, which accounted for the remaining resistance that was not efflux-mediated. Expression analysis of the genes coding for the main efflux pumps revealed increased expression only in the presence of inducing agents. Moreover, it showed that not only different substrates can trigger expression of different EP genes, but also that the same substrate can promote a variable response, according to its concentration. We also found isolates belonging to the same clonal type that showed different responses towards drug exposure, thus evidencing that highly related clinical isolates may diverge in the efflux-mediated response to noxious agents. The data gathered by real-time fluorometric and RT-qPCR assays suggest that *S. aureus *clinical isolates may be primed to efflux antimicrobial compounds.

**Conclusions:**

The results obtained in this work do not exclude the importance of mutations in resistance to fluoroquinolones in *S. aureus*, yet they underline the contribution of efflux systems for the emergence of high-level resistance. All together, the results presented in this study show the potential role played by efflux systems in the development of resistance to fluoroquinolones in clinical isolates of *S. aureus*.

## Background

*Staphylococcus aureus *infections, particularly those caused by methicillin-resistant *S. aureus *(MRSA), pose serious therapeutic difficulties and are a major concern in both the nosocomial and community settings. The use of fluoroquinolones for the effective treatment of these infections is impaired by the swift emergence of fluoroquinolone resistance, a trait widely spread among clinical MRSA strains [1,2].

Fluoroquinolone resistance in *S. aureus *has been mainly attributed to mutations occurring in the quinolone-resistance determining region (QRDR) of GrlA/GrlB (topoisomerase IV, encoded by genes *grlA/grlB*) and GyrA/GyrB (DNA gyrase, encoded by genes *gyrA/gyrB*); which decrease their affinity to the drug [3-5]. However, fluoroquinolone resistance can also be mediated by drug efflux, a mechanism that is less well characterized [6].

To date, several efflux pumps (EPs) have been described for *S. aureus*, including the chromosomally encoded NorA, NorB, NorC, MdeA, MepA, SepA and SdrM, as well as the plasmid-encoded QacA/B, QacG, QacH, QacJ and Smr [7]. Whereas these efflux pumps show different substrate specificity, most of them are capable of extruding compounds of different chemical classes. These features reveal the potential role of EPs in providing the cell with the means to develop a multidrug resistance (MDR) phenotype and consequently survive in hostile environments.

A variety of methods have been used to identify active efflux systems in bacteria, such as the use of radiolabelled substrates, fluorometric assays or the determination of the minimum inhibitory concentration (MIC) for different substrates in the presence of compounds known to modulate the activity of efflux pumps (usually described as efflux inhibitors, EIs) [8-10]. This work aimed to assess and characterize the presence of active efflux systems in clinical isolates of *S. aureus *using several methodologies and to understand their role in the development of resistance to fluoroquinolones by *S. aureus *in the clinical setting, since fluoroquinolones are considered substrates of the majority of the pumps encoded by the *S. aureus *chromosome [7].

## Results

### Detection of active efflux systems by the Ethidium Bromide (EtBr)-agar Cartwheel (EtBrCW) Method

For this study, we selected all the *S. aureus *isolates presenting resistance towards ciprofloxacin received by the Bacteriology Laboratory of one of the largest hospitals in Portugal during a four months period. These corresponded to a collection of 52 *S. aureus *isolates.

Efflux activity amongst these 52 ciprofloxacin resistant isolates was assessed by means of a fast and practical test, the Ethidum Bromide-agar Cartwheel (EtBrCW) Method that provides information on the capacity of each isolate to extrude EtBr from the cells by efflux, on the basis of the fluorescence emitted by cultures swabbed in EtBr-containing agar plates. Those cultures showing fluorescence at lower EtBr concentrations have potentially less active efflux systems than those for which fluorescence is only detected at higher concentrations of EtBr [11,12]. The application of this method allowed the selection of 12 *S. aureus *isolates showing increased EtBr efflux activity when compared to the non-effluxing control strain ATCC25923 and to the efflux-positive control strain ATCC25923_EtBr _[13]. These 12 isolates were designated EtBrCW-positive isolates, whereas the remaining 40 isolates were considered to have no or intermediate efflux activity and therefore designated as EtBrCW-negative isolates (Table 1).

**Table 1:** Genotypic and phenotypic characterization of *S. aureus *clinical isolates.

		QRDR mutations^b^		MIC (mg/L)^c^											
		
				EtBr			CIP			NOR			NAL		
				
Isolate^a^	PFGE pattern	GrlA	GyrA	No	+	+	No	+	+	No	+	+	No	+	+
				EI	TZ	CPZ	EI	TZ	CPZ	EI	TZ	CPZ	EI	TZ	CPZ
ATCC25923	-	WT	WT	6.25	**0.75**	**0.75**	0.25	0.125	0.125	0.5	**0.125**	**0.125**	64	n.d.	n.d.
ATCC25923_EtBr_	-	WT	WT	200	**25**	**12.5**	1	**0.25**	**0.25**	2	**0.25**	**0.25**	64	n.d.	n.d.
**SM1**	A2	S80Y/E84K	S84L	16	**4**	**4**	128	**32**	64	512	**128**	256	256	**64**	**64**
**SM10**	A4	S80Y/E84K	S84L	16	**2**	**4**	128	64	64	512	**128**	**128**	128	64	64
**SM14**	A3	S80Y/E84K	S84L	16	**4**	**4**	256	**32**	128	1024	**128**	**256**	256	**64**	**64**
**SM17**	A4	S80Y/E84K	S84L	16	**4**	**4**	256	**64**	**64**	1024	**256**	512	256	**64**	**64**
**SM25**	A1	S80Y/E84K	S84L	8	**2**	4	128	**32**	64	512	**64**	**128**	256	**32**	64
**SM27**	A4	S80Y/E84K	S84L	16	**4**	**4**	256	**32**	**64**	512	**128**	256	256	**64**	**64**
**SM43**	A1	S80Y/E84K	S84L	16	**2**	**4**	128	64	64	512	**128**	**128**	512	256	**64**
**SM46**	A1	S80Y/E84K	S84L	16	**4**	**4**	128	64	64	512	**128**	256	128	64	64
**SM47**	A1	S80Y/E84K	S84L	8	**2**	4	256	**32**	**64**	512	**128**	256	256	**64**	**64**
**SM48**	A1	S80Y/E84K	S84L	8	4	4	256	**32**	**64**	512	**128**	256	256	**64**	**64**
**SM50**	B1	S80F/E84K	S84L	8	**1**	**2**	64	**16**	**16**	256	**32**	**64**	128	64	64
**SM52**	C1	E84K	S84L	16	**1**	**2**	16	8	8	64	32	32	128	**32**	64
SM2	B2	S80F/E84K	S84L	8	**2**	**2**	32	16	16	128	**32**	**32**	64	**16**	64
SM3	E1	S80Y/E84K	S84L	1	1	1	16	8	8	64	32	32	64	**16**	**16**
SM4	E2	S80F	S84L	4	2	**1**	8	8	8	64	32	32	64	32	64
SM5	E3	S80Y/E84K	S84L	4	2	**1**	32	16	16	128	64	64	64	32	32
SM6	A5	S80F	E88K	4	2	**1**	16	16	16	64	32	32	64	32	32
SM7	E1	S80F	S84L	2	2	1	8	8	4	64	32	32	128	**32**	64
SM8	A5	S80F	E88K	4	2	**1**	16	8	16	128	64	64	128	**32**	64
SM12	E1	S80F	S84L	2	2	1	16	8	8	64	32	32	128	**32**	64
SM16	A6	S80F	E88K	4	2	**1**	16	16	16	128	**32**	64	64	32	64
SM22	A1	S80Y/E84G	S84L	8	4	4	128	**16**	**32**	512	**128**	**128**	64	32	64
SM34	D1	S80F/E84K	S84L	4	2	2	64	**16**	32	64	**16**	32	32	16	32
SM36	E1	S80F	S84L	4	2	2	16	8	8	64	**16**	32	128	**32**	64
SM40	E1	S80F	S84L	8	4	4	32	32	32	512	**128**	**128**	16	8	16

^a^Isolates in bold correspond to the EtBrCW-positive isolates. ^b^WT: wild-type; S: serine; F: phenylalanine; E: glutamate; K: lysine; Y: tyrosine; L: leucine; G: glycine. ^c^Values in bold-type correspond to a MIC decrease of ≥ four-fold in the presence of the efflux inhibitor (EI) in comparison to the values with no EI [10]. The concentration of each EI used is defined in the Methods section. EtBr: ethidium bromide; CIP: ciprofloxacin; NOR: norfloxacin; NAL: nalidixic acid; TZ: thioridazine; CPZ: chlorpromazine; n.d.: not determined.

Based upon these results, we continued the study by further analyzing the 12 EtBrCW-positive isolates, as well as a group of representative 13 EtBrCW-negative isolates, as controls.

### Real-time assessment of efflux activity

In order to characterize the efflux activity of the cells, we used a semi-automated fluorometric method previously developed by our group [14], which allows monitoring, on a real-time basis, the accumulation of EtBr inside the bacterial cells, followed by its efflux.

**Figure 1:**
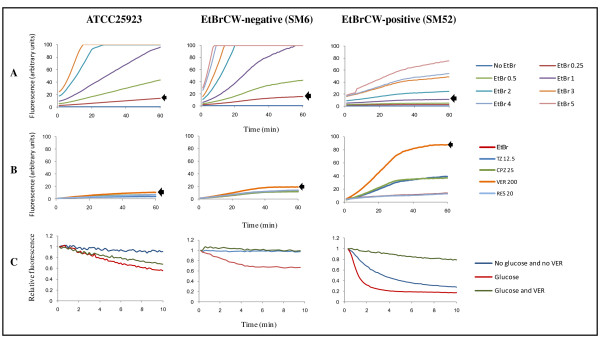
**Real-time EtBr accumulation/efflux for the representative strains ATCC25923 (reference), SM6 (EtBrCW-negative) and SM52 (EtBrCW- positive)**. **Panel A: Assessment of EtBr accumulation**. The arrow indicates the EtBr accumulation at the concentration (mg/L) chosen for the subsequent assays (panels B and C). **Panel B: Assessment of EtBr accumulation in the presence of efflux inhibitors**. The EIs were tested at a sub-inhibitory concentration, namely TZ: thioridazine (12.5 mg/L); CPZ: chlorpromazine (25 mg/L); VER: verapamil (200 mg/L) and RES: reserpine (20 mg/L). The arrow indicates the EtBr accumulation in the presence of the most effective EI for each isolate. **Panel C: Assessment of EtBr efflux**. The assays were done in the presence/absence of 0.4% glucose, with or without the EI verapamil (VER) at a sub-inhibitory concentration of 200 mg/L. The data presented was normalized against the data obtained in conditions of no efflux (absence of glucose and presence of 200 mg/L of VER).

The first step of this technique is to establish the ideal conditions for EtBr accumulation inside the cells. Thus, assays were initially performed to determine the EtBr concentration above which there is detectable accumulation and to select the most effective efflux inhibitor; that is the EI that promotes the highest EtBr accumulation. The EtBr accumulation assays showed that the two groups of isolates previously established by the EtBrCW Method differed with respect to their capacity to accumulate EtBr, with EtBrCW-negative isolates retaining more EtBr than the EtBrCW-positive isolates (Figure 1-A). The same result was observed for the reference strain ATCC25923. These differences were reflected in the minimum EtBr concentration required for detectable accumulation, which was higher for the EtBrCW-positive isolates. The accumulation assays performed in the presence of several EIs showed that verapamil was the most effective in promoting accumulation of EtBr, for either EtBrCW-positive isolates, EtBrCW-negative isolates or the reference strain (Figure 1-B).

The conditions established by the accumulation assays were then used to load cells with EtBr and perform efflux assays. The assessment of EtBr efflux on a real-time basis (during a 10 min frame) detected a considerable difference between EtBrCW-positive isolates, which showed a pronounced efflux pump activity, with a prompt and significant decrease in fluorescence and the EtBrCW-negative isolates, that showed only basal efflux pump activity, similar to the one presented by the reference strain (Figure 1-C). These results confirm the presence of increased efflux activity in the EtBrCW-positive isolates relatively to the EtBrCW-negative isolates.

### Effect of efflux inhibitors on MICs of fluoroquinolones and EtBr

As expected, since all clinical isolates were selected on the basis of resistance to ciprofloxacin, they all presented high MIC values for fluoroquinolones. Nevertheless, the majority of the EtBrCW-positive isolates displayed higher MIC values for the fluoroquinolones tested and EtBr, whilst the EtBrCW-negative isolates presented significantly lower values, although some overlap exists between the two sets of MIC values (Table 1). The EIs reduced the MIC values for fluoroquinolones and EtBr of the EtBrCW-positive isolates to the values presented by the EtBrCW-negative isolates, confirming the presence of an active efflux component in those isolates (Table 1). The EIs thioridazine (TZ) and chlorpromazine (CPZ) were the most effective in reducing the MIC values. Verapamil (VER) and reserpine (RES) showed a smaller or absent inhibitory effect, while carbonyl cyanide *m*-chlorophenylhydrazone (CCCP) showed no effect on the MIC values for the compounds tested (data not shown). However, no full reversion of the fluoroquinolone resistance phenotype was obtained with any of the EIs tested, suggesting the contribution of other mechanisms to this resistance, namely, mutations in the target genes.

### Screening for mutations conferring fluoroquinolone resistance

The 25 isolates representing both EtBrCW-positive and negative isolates were screened for the presence of chromosomal mutations most commonly associated with fluoroquinolone resistance in *S. aureus*, namely the ones occurring in the QRDRs of both *grlA *and *gyrA *genes [3,5,15,16]. All isolates tested carried mutations in *grlA *and *gyrA *related to fluoroquinolone resistance, in six different combinations at the protein level (Table 1). The majority of the isolates presented a double mutation in GrlA together with a single mutation in GyrA, with 12 isolates carrying the GrlA and GyrA mutations S80Y/E84K and S84L, respectively; three isolates carrying mutations GrlA S80F/E84K and GyrA S84L; and one isolate carrying mutations GrlA S80Y/E84G and GyrA S84L. The other nine isolates screened showed a single mutation in both GrlA and GyrA, in three distinct arrangements (Table 1).

The overall analysis of these results reveals a clear distinction between the EtBrCW-positive and the EtBrCW-negative isolates, with each group showing a relatively homogeneous profile, both in terms of efflux capacity and mutations in the genes related to fluoroquinolone resistance. In order to test if such homogeneity would be the result of clonal expansion of specific *S. aureus *clones, the isolates were then typed by macrorestriction analysis.

### Macrorestriction analysis

**Figure 2:**
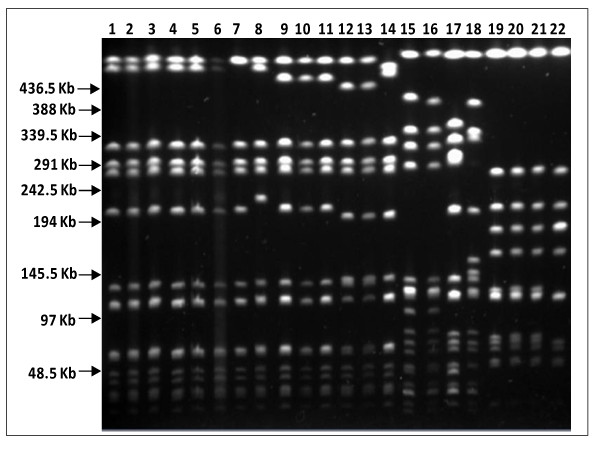
***Sma*I macrorestriction profiles of *S. aureus *clinical isolates**. Numbers correspond to the following isolates: 1- SM43; 2- SM46; 3- SM47; 4- SM48; 5- SM22; 6- SM25; 7- SM1; 8- SM14; 9- SM10; 10- SM17; 11- SM27; 12- SM6; 13- SM8; 14- SM16; 15- SM50; 16- SM2; 17- SM52; 18- SM34; 19- SM36; 20- SM40; 21- SM3; 22- SM4. The arrows show the position and weight of the lambda ladder molecular size marker.

The clonality of the *S. aureus *clinical isolates was assessed by pulsed-field gel electrophoresis (PFGE) analysis of *Sma*I macrorestriction profiles. According to the criteria of Tenover *et al *[17], six clones were found among the entire collection. The two predominant clones, A and E, included several sub-clones and comprised 25 and 18 isolates, respectively. The remaining clones B, C, D and F, were represented by 1 to 6 isolates (representative data is presented in Table 1 and Figure 2).

Of the 12 EtBrCW-positive isolates, 10 belonged to clone A, one to clone B and one to clone C. On the other hand, the 40 EtBrCW-negative isolates included all isolates from clone E (18 isolates) plus isolates from clone A (15), clone B (5), clones D and F (1 isolate each).

### Expression analysis of *S. aureus *efflux pump genes

The presence of EP genes was assessed by PCR. All *S. aureus *isolates carried the five chromosomal genes tested (*norA*, *norB*, *norC*, *mepA *and *mdeA*) and one isolate, SM52, carried the plasmid encoded *smr *gene, whereas no isolate was found to carry the plasmid encoded *qacA/B *gene.

To assess the contribution of each individual pump to the overall efflux activity presented by each strain, ten isolates representative of each clone or sub-clone (six EtBrCW-positive and four EtBrCW-negative,) plus reference strain ATCC25923 (also EtBrCW-negative), were selected for expression analysis by RT-qPCR of EP genes.

When determining the expression level of efflux pump genes for the clinical isolates in comparison to the reference strain ATCC25923 in drug-free media, no overexpression of these genes was detected (data not shown). When these clinical isolates and ATCC25923 were exposed to an efflux pump substrate, either ciprofloxacin or EtBr, at ½ their MICs, and gene expression levels determined against the respective unexposed condition, overexpression of efflux pump genes was detected in six clinical isolates, three EtBrCW-negative and three EtBrCW-positive as well as in the reference strain itself (Table 2).

**Table 2:** EP gene expression analysis by RT-qPCR of representative *S. aureus *exposed to CIP or EtBr.

	Overexpression levels* and no. of isolates** showing gene overexpression					
	
	½ CIP MIC			½ EtBr MIC		
	
		EtBrCW-	EtBrCW+		EtBrCW-	EtBrCW+
Gene	ATCC25923	isolates	isolates	ATCC25923	isolates	isolates
		(n = 4)	(n = 6)		(n = 4)	(n = 6)
***norA***	-	-	-	4.51 ± 0.77	-	-
		0	0		0	0

***norB***	13.80 ± 6.50	5.43 **± **2.39	5.47 **± **0.19	7.07 ± 2.78	5.33 **± **0.73	-
		**2^a, b^**	**1^e^**		**1^a^**	0

***norC***	-	-	4.92 **± **0.00	5.89 ± 0.71	4.99 **± **1.51	-
		0	**1^e^**		**1^a^**	0

***mepA***	-	-	8.59 **± **0.59	3.90 ± 0.13	5.94 **± **1.02	-
		0	**1^f^**		**1^a^**	0

***mdeA***	-	4.97 **± **0.68	-	3.96 ± 2.10	-	4.15 **± **1.12
		**1^c^**	0		0	**1^d^**

***smr***	n.a.	n.a.	-	n.a.	n.a.	7.66 **± **3.66
			0			**1^f^**

* Gene expression was measured in the presence of ciprofloxacin and EtBr relatively to the drug-free condition. The results are expressed in terms of the mean **± **standard deviation of at least three independent assays performed with independently extracted RNAs and correspond to the range of values obtained for isolates showing overexpression of that gene. **The numbers in bold correspond to the number of isolates overexpressing that gene: ^a ^isolate SM2; ^b ^SM3; ^c ^SM5; ^d^SM25; ^e ^SM50; ^f ^SM52. Overexpression was considered for values ≥4 [10]. (-): no overexpression was detected; n.a.: not applicable.

The majority of the isolates showed overexpression of a single efflux pump gene, most frequently, *norB *or *mdeA*. One isolate showed overexpression of two efflux pump genes (*norB/norC*) and another one overexpressed three EP genes (*norB*/*norC*/*mepA*). Overall, isolates showed to be more responsive to ciprofloxacin. The *smr *gene was found to be overexpressed only in the presence of EtBr, in accordance to the substrate specificity described in the literature for this pump [18]. These same agents had a distinct effect on ATCC25923, which showed significant overexpression of all efflux pump genes tested in the presence of EtBr, and a higher overexpression of *norB *when exposed to ciprofloxacin (Table 2).

The effect of drug exposure on the expression level of the efflux pump genes was further explored by increasing the ciprofloxacin concentration to ¾ the MIC. Isolates that showed EP gene overexpression with ½ the MIC of ciprofloxacin showed either an increase in that expression level or the overexpression of additional genes. For instance, EtBrCW-positive isolate SM50 overexpressing *norB*/*norC *with ½ MIC of ciprofloxacin, now showed even higher expression of *norB *(37.05 ± 18.67) and *norC *(83.98 ± 19.98) and *de novo *overexpression of *norA *(8.36 ± 4.63) and *mepA *(45.86 ± 13.86). Likewise, exposure of the EtBrCW-negative SM2 to higher ciprofloxacin concentrations resulted in increased levels of *norB *expression (4.48 ± 2.48) that was accompanied by *de novo *overexpression of *norC *(5.33 ± 0.73) and *mepA *(10.58 ± 0.73).

## Discussion

The few studies on efflux among *S. aureus *clinical isolates use the decrease of antibiotic MICs in the presence of EIs, particularly reserpine, as indicative of efflux activity [10]. This approach is laborious and dependent on the susceptibility of the efflux system(s) to reserpine, which varies considerably [19]. More recently, Patel and colleagues have proposed the use of EtBr MICs to identify *S. aureus *effluxing strains [20]. This approach has the advantage of assessing efflux activity using a broad range efflux pump substrate, EtBr, which is pumped out by most efflux systems described for *S. aureus*, and thus, is an useful marker for the detection of efflux pump activity [7,12,14,20]. Nevertheless, it is still an indirect assessment of efflux activity.

In the present study, we have applied two methods for the direct assessment of efflux activity among a collection of 52 ciprofloxacin resistant *S. aureus *clinical isolates, both also based on EtBr efflux. We first applied the EtBr-agar Cartwheel Method to select isolates with increased efflux activity. The presence of increased efflux in the 12 isolates selected was supported by the data collected from the semi-automated fluorometric method, which demonstrated that EtBrCW-positive isolates had a higher efflux activity than the EtBrCW-negative isolates. Thus, both methods proved to be adequate to assay efflux activity in *S. aureus *cells. In particular, the EtBrCW method proved to be a valuable tool for the rapid screening of efflux pump activity, allowing its application to screen large collections of clinical isolates. Furthermore, the use of a broad range efflux pump substrate such as EtBr warrants its wider application as compared to the analysis of EIs effect on MIC values, which can be severely impaired by the susceptibility of each efflux system to the EI being used and for which the mechanism of action at the cellular level remains, in most cases, to be clarified.

In addition, the semi-automated fluorometric method also allowed the characterization of this efflux activity, in terms of maximal concentration of EtBr that the cells were able to extrude without observable accumulation over a 60 min period and susceptibility toward several EIs. The results obtained clearly showed a distinct capacity of the two groups of isolates to extrude EtBr from their cells, with the EtBrCW-positive isolates being able to handle higher EtBr concentrations with no detectable accumulation. It was also observed that for both groups of isolates, EtBr extrusion/accumulation was most affected by the EI verapamil.

The efflux assays further demonstrated the higher efflux capacity of the EtBrCW-positive isolates, with a pronounced decreased of EtBr fluorescence (80%) within a 2 min interval, whereas the EtBrCW-negative isolates showed a smaller decrease of EtBr fluorescence (40%) over a 10 min interval.

These results were then complemented with MIC determination in the presence of EIs, leading to the observation that the efflux-mediated resistance is an important component of the level of fluoroquinolone resistance. In fact, not only the 12 EtBrCW-positive isolates presented higher MIC values towards the several fluoroquinolones, also these MIC decreased to levels similar to those of the EtBrCW-negative isolates in the presence of TZ and CPZ, even for isolates sharing the same QRDR mutations (Table 1). Altogether, these data demonstrate that mutations in the QRDR of *grlA *and *gyrA *genes confer resistance up to a certain level (8-32 mg/L for ciprofloxacin), above which resistance is mainly efflux-driven. This implies that although the inhibition of the efflux component by EIs does not bring resistance down to the susceptibility level, it promotes a significant decrease in this resistance.

In the MIC assays TZ and CPZ were the two EIs with the highest effect, whereas in the fluorometric assay, EtBr extrusion/accumulation was most affected by verapamil. This should reflect differences in the mechanism of action of each molecule, as well as to the characteristics of each assay. We have recently observed the same type of results with isolates of *Mycobacterium smegmatis *[21]. The absence of efflux inhibitory effect of CCCP at sub-MIC concentrations for *S. aureus *strains has been discussed in a previous study [13].

For the analysis of gene expression, we first compared our clinical isolates to a fully-antibiotic susceptible reference strain, *S. aureus *ATCC25923, following the rationale of previous studies, [10,20,22]. However, in contrast to these earlier studies, no EP gene was found to be overexpressed. Consequentially, we explored the effect of exposing the isolates to ½ the MIC of the antimicrobial compounds used previously as selective markers, ciprofloxacin and EtBr, using the isolates grown in a drug-free condition as a reference for determining the gene expression level. Using this approach, we were able to detect overexpression of EP genes, albeit at levels lower than the ranges described in literature [10,20,22]. These differences could, in some extent, reflect the different approaches used, including the use of a different reference strain for gene expression assays. Nevertheless, the different methodological approaches do not explain all the results and since EtBrCW-positive isolates showed a strong involvement of efflux in the resistance phenotype, the absence of high levels of efflux pump genes expression suggests that the isolates could be already primed to respond to these noxious compounds. Clinical isolates are under a constant pressure by antimicrobial compounds, such as antibiotics and biocides. Since the expression of efflux pumps provides the cell with the means to cope with these compounds, it could be expected that those clinical isolates already have in their cell membrane the necessary number of efflux pump proteins, thus, increases in efflux pump genes expression may have already taken place. Also, no significant differentiation could be established between EtBrCW-positive and EtBrCW-negative isolates at the level of individual EP gene expression (Table 2). On the other hand, ATCC25923, which showed only basal efflux activity on the fluorometric assay, responded to drug pressure in a completely different manner, showing a significant overexpression of all efflux pump genes tested in the presence of EtBr and the highest expression level of *norB *following exposure to ciprofloxacin (Table 2). The distinct behavior observed for the clinical isolates as compared to the antibiotic fully susceptible reference strain further support the hypothesis that the clinical strains are primed to efflux noxious substances.

Increasing the concentration of ciprofloxacin to ¾ of the MIC augmented the expression rate of the already overexpressed genes with the additional overexpression of other efflux pump genes. These results show a clear concentration level above which there is an inducement of expression of the same or additional efflux pump genes. This response could reflect the involvement of these genes in a global stress response regulon, or simply be the result of a substrate-responsive regulation. Future work should clarify this aspect.

A previous study described the predominance of *norB *overexpression among a collection of *S. aureus *bloodstream isolates. For this collection, when a single efflux pump gene was overexpressed, it corresponded mostly to *norA*, whereas *norB *and *norC *were prevalent when two or more efflux pump genes were overexpressed [10]. In our work, amongst the clinical isolates that overexpressed efflux pump genes, four showed overexpression of a single gene, either *norB*, *mdeA *or *mepA*. Only two isolates showed overexpression of more than one efflux pump gene. Remarkably, *norA *was the only gene for which no overexpression was detected among the clinical isolates, suggesting that other efflux pumps can have a more relevant role in the resistance to fluoroquinolones and EtBr in *S. aureus *than the one attributed to date. Nevertheless, exposure of ATCC25923 to EtBr, resulted in the overexpression of all efflux pump genes tested, including *norA*. This result does not oppose to our previous finding that the prolonged exposure of this strain to increasing concentrations of EtBr resulted in high overexpression of solely *norA *[13], inasmuch as it strengthens the premise that exposure of the same strain to a given drug over different ranges of concentrations and/or time may result in the activation of different efflux systems. Our data also revealed that the same clinical isolate can respond differently at the gene expression level, to the presence of two inducers, ciprofloxacin and ethidium bromide, both common substrates of the main multidrug efflux pumps in *S. aureus*. For example, the EtBrCW-negative isolate SM2 exposed to ciprofloxacin showed only *norB *overexpression, whilst in the presence of EtBr, it overexpressed *norB*, *norC *and *mepA*. In the particular case of strain SM52, the plasmid encoded Smr pump was only overexpressed upon exposure to EtBr, whereas when challenged with ciprofloxacin, the strain responded with the overexpression of *mepA*. Our data also demonstrates that isolates from the same clone, as defined by PFGE, can have distinct levels of efflux activity and respond to the same agent through the activation of different efflux pumps (cf Tables 1 and 2).

## Conclusions

The rationale and methodologies applied in this study showed that efflux activity is an important component of the resistance to fluoroquinolones and other compounds in clinical isolates of *S. aureus*. We demonstrated that not only different substrates can trigger different pumps, but also that the same substrate can promote a variable response, according to its concentration, thus strengthening the crucial role played by efflux pumps in the survival of *S. aureus *clinical isolates in health-care settings. Additionally, our study underlines the importance of using new molecular approaches to fully understand the function that each individual efflux pump undertakes in the bacterial cell response to antimicrobial compounds.

In particular, although specific clones could be found among either EtBrCW-positive or EtBrCW-negative bacteria, isolates belonging to the same clonal type showed different responses towards drug exposure, thus evidencing that highly related clinical isolates, sharing the same genetic background, may diverge in the efflux-mediated response to noxious compounds. The data gathered by the semi-automated fluorometric method together with the results from the RT-qPCR assays, sustain the hypothesis that *S. aureus *clinical isolates may be primed to efflux antimicrobial compounds. Therefore, the lack of a marked response to the induction of efflux pump genes expression may be explained by the higher efflux capacity already present in all the clinical isolates tested, when compared to the naive reference strain *S. aureus *ATCC25923.

Altogether, the results presented in this study show the potential role played by efflux systems in the development of resistance to fluoroquinolones in hospitals and the contribution of the several *S. aureus *efflux systems to this resistance.

## Methods

### Bacterial isolates

#### Reference strains

*S. aureus *strain ATCC25923, a clinical isolate collected at Seattle in 1945 and ATCC25923_EtBr _[13], belonging to the culture collection of the Grupo de Micobactérias, Unidade de Microbiologia Médica, Instituto de Higiene e Medicina Tropical (IHMT/UNL), were used as controls.

#### Clinical strains

A collection of 52 *S. aureus *was studied, comprising all the ciprofloxacin resistant clinical isolates sampled at the Bacteriology Laboratory of a 1, 300-bed teaching hospital (Lisbon, Portugal), from December 2006 to March 2007. These corresponded to 49 MRSA and 3 MSSA, isolated from single patients and different biological products. All isolates were tested for identification and antibiotic susceptibility by the automated system WalkAway^® ^(Dade Behring™) and selected on the basis of their resistance to ciprofloxacin.

### Growth conditions

Strains were grown in tryptic soy broth (TSB) at 37°C with shaking or in tryptic soy agar (TSA) (Oxoid Ltd., Basingstoke, UK). Strain ATCC25923_EtBr _was grown in TSB or TSA supplemented with 50 mg/L of EtBr. For determination of minimum inhibitory concentrations (MICs), cultures were grown in Mueller-Hinton broth (MH, Oxoid) at 37°C.

### Antibiotics and dyes

Antibiotics in powder form were purchased from different sources, as follows: nalidixic acid (Sigma-Aldrich, St. Louis, MO, USA); norfloxacin (ICN Biomedicals Inc., Ohio, USA); ciprofloxacin (Fluka Chemie GmbH, Buchs, Switzerland). EtBr was acquired in powder form from Sigma (Madrid, Spain).

### Efflux inhibitors (EIs)

Carbonyl cyanide *m*-chlorophenylhydrazone (CCCP), thioridazine (TZ), chlorpromazine (CPZ), verapamil (VER) and reserpine (RES) were purchased from Sigma. Solutions of TZ, CPZ and VER were prepared in desionized water; RES was prepared in dimethylsulfoxide (DMSO) and CCCP in 50% methanol (v/v). All solutions were prepared on the day of the experiment and kept protected from light.

### EtBr-agar Cartwheel (EtBrCW) Method

This simple method tests the presence of active efflux systems [11,12,23], being an update of the already described, EtBr-agar screening method [23,24]. It provides information on the capacity of each isolate to extrude EtBr from the cells by efflux pumps, on the basis of the fluorescence emitted by cultures swabbed in EtBr-containing agar plates. Briefly, each culture was swabbed onto TSA plates containing EtBr concentrations ranging from 0.5 to 2.5 mg/L (0.5 mg/L increments). *S. aureus *cultures ATCC25923 and ATCC25923_EtBr _were used as negative and positive controls for efflux activity, respectively [13]. The plates were incubated at 37°C during 16 hours, after which the minimum concentration of EtBr associated with the bacterial mass that produced fluorescence under UV light was recorded in a Gel-Doc XR apparatus (Bio-Rad, Hercules, CA, USA). Isolates showing fluorescence at lower EtBr concentrations have potentially less active efflux systems than isolates for which fluorescence is only detected at higher concentrations of EtBr [11,12,23,24]. Isolates showing emission of fluorescence only at the maximum concentration of EtBr tested (2.5 mg/L) were considered to have potential active efflux systems.

### Drug susceptibility testing

#### Antibiotics and EtBr

MICs for antibiotics were determined by the two-fold broth microdilution method [25]. Results were evaluated according to the CLSI breakpoints [25], except for nalidixic acid, for which there are no defined breakpoints. MICs for EtBr were also determined using the two-fold broth microdilution method. After an 18 hour incubation period at 37°C, the MIC values were recorded, corresponding to the lowest concentration of EtBr that presented no visible growth. All MICs were determined in triplicate.

#### Efflux inhibitors (EIs)

Each EI employed in this study was evaluated for its ability to reduce or reverse resistance to given antibiotics or EtBr, both of which are characteristics that define the agent as an inhibitor of efflux pump activity [26]. The evaluation of an agent for EI activity was conducted in medium containing varying concentrations of the antibiotic or EtBr and a bacterial inoculum corresponding to the one used for MIC determination. Parallel cultures were tested in media containing no EI and EI (at sub-lethal concentrations, see below) plus varying concentrations of the compound to be tested. The cultures were incubated for 18 hours and growth evaluated visually. An EI was considered to have an inhibitory effect when a decrease of at least four-fold in the MIC was observed in the presence of that EI, relatively to the original MIC [10]. MICs of each EI were determined by the two-fold broth microdilution method, as described above. The final concentrations of the EIs used, which correspond to half, or below, the MICs determined for each EI, were: TZ (12.5 mg/L); CPZ (25 mg/L); VER (200 mg/L); RES (20 mg/L) and CCCP (0.25 mg/L). All assays were performed in triplicate.

### Semi-automated fluorometric method

This method allows the real-time fluorometric detection of the accumulation of a given efflux pump substrate (in this case, EtBr) inside cells and its efflux, using a Rotor-Gene 3000™ thermocycler, together with real-time analysis software (Corbett Research, Sydney, Australia) [14,27,28]. Accumulation assays allow to assess the EtBr concentration above which detectable EtBr accumulation occurs and to select the most effective efflux inhibitor; that is the EI that promotes the highest EtBr accumulation [14]. These conditions can then be used to load bacterial cells with EtBr and follow its efflux.

For the accumulation assays, the cultures were grown in TSB medium at 37°C with shaking until they reach an optical density at 600 nm (OD_600 nm_) of 0.6. To prepare the cellular suspension, the cells were collected by centrifugation at 13, 000 rpm for 3 minutes and the pellet washed twice with a 1X Phosphate Buffered Saline (PBS) solution. The OD_600 nm _of the cellular suspension was then adjusted to 0.6 in 1X PBS. To determine the EtBr concentration where there is detectable accumulation, several assays were prepared in 0.1 mL (final volume) containing 0.05 mL of the cellular suspension (final OD_600 nm _of 0.3) and 0.05 mL of 2X EtBr stock solutions (final concentrations of 0.25, 0.5, 1, 2, 3, 4 and 5 mg/L). To determine the most effective EI, assays were prepared in a final volume of 0.1 mL containing 0.05 mL of the cellular suspension (final OD_600 nm _0.3) and 0.05 mL of a solution containing 2X the EtBr concentration previously selected and 2X the EI concentration to be tested (final concentrations of TZ: 12.5 mg/L, CPZ: 25 mg/L, VER: 200 mg/L, RES: 20 mg/L). All assays included control tubes containing only the isolate (0.05 mL of cellular suspension at OD_600 nm _of 0.6 plus 0.05 mL of 1X PBS) and only the EtBr concentration to be tested (0.05 mL of 2X EtBr stock solution plus 0.05 mL of 1X PBS). The assays were then run in a Rotor-Gene 3000™ at 37°C, and the fluorescence of EtBr was measured (530/585 nm) at the end of every cycle of 60 seconds, for a total period of 60 minutes.

For the efflux assays, EtBr-loaded cells were prepared by incubating a cellular suspension with an OD_600 nm _of 0.3 with either 0.25 or 1 mg/L EtBr for EtBrCW-negative or positive cultures, respectively and 200 mg/L VER at 25°C for 60 minutes. After EtBr accumulation, cells were collected by centrifugation and re-suspended in 1X PBS to an OD_600 nm _of 0.6. Several parallel assays were then run in 0.1 mL final volume corresponding to 0.05 mL of the EtBr loaded cells (final OD_600 nm _of 0.3) incubated with 0.05 mL of (1) PBS 1X only; (2) glucose 0.8% only (final concentration of 0.4%); (3) 2X VER only (final concentration of 200 mg/L); (4) glucose 0.8% (final concentration of 0.4%) plus 2X VER (final concentration of 200 mg/L). These efflux assays were conducted in the Rotor-Gene 3000™ at 37°C, and the fluorescence of EtBr was measured (530/585 nm) at the end of every cycle of 10 seconds, for a total period of 10 minutes. The raw data obtained was then normalized against data obtained from non-effluxing cells (cells from the control tube with only 200 mg/L VER), at each point, considering that these correspond to the maximum fluorescence values that can be obtained during the assay. The relative fluorescence thus corresponds to the ratio of fluorescence that remains per unit of time, relatively to the EtBr-loaded cells.

### Macrorestriction analysis

Isolates were typed by pulsed-field gel electrophoresis (PFGE) analysis, using well-established protocols. Briefly, agarose disks containing intact chromosomal DNA were prepared as previously described [29] and restricted with *Sma*I (New England Biolabs, Ipswich, MA, USA), according to the manufacturer's recommendations. Restriction fragments were then resolved by PFGE, which was carried out in a contour-clamped homogeneous electric field apparatus (CHEF-DRIII, Bio-Rad), as previously described [29]. Lambda ladder DNA (New England Biolabs) was used as molecular weight marker. PFGE types were defined according to the criteria of Tenover *et al*. [17].

### Preparation of chromosomal DNA

Genomic DNA was extracted with the QIAamp DNA Mini Kit (QIAGEN, Hilden, Germany), with an additional step of 30 minutes digestion with lysostaphin (Sigma) (200 mg/L) prior to extraction.

### Preparation of plasmid DNA

The QIAprep Spin Miniprep kit (QIAGEN) was used, with the following modification: prior to extraction, cells were incubated with lysostaphin (35 mg/L) at 37°C for 90 minutes, as previously described [30].

### Screening of mutations in *grlA *and *gyrA *genes

Internal fragments comprising the QRDR of *grlA *and *gyrA *genes were amplified using the primers described in Table 3. The reaction mixture (50 μL) contained 2.5 U of *Taq *Polymerase (Fermentas Inc., Ontario, Canada), 1X *Taq *buffer (Fermentas); 25 pmol of each primer; 0.2 mM of dNTP and 1.75 mM of MgCl_2_. The PCR reactions were conducted in a thermocycler Mastercycler personal 5332 (Eppendorf AG, Hamburg, Germany). The amplification conditions were as follows: DNA was denatured at 94°C for 4 minutes, followed by 35 cycles of denaturation at 94°C for 30 seconds, annealing at 50°C for 30 seconds and extension at 72°C for 1 minute, followed by a step of final extension at 72°C for 5 minutes. Amplification products were purified and sequenced in both strands using the same set of primers. Sequences were analyzed and aligned using the freeware programs BioEdit and ClustalW, respectively.

**Table 3:** Primers used in this study.

Primer^a^	Sequence (5'-3')	Amplicon Size (bp)	Reference
QacA/B_Fw	GCTGCATTTATGACAATGTTTG	628	[30]
QacA/B_Rv	AATCCCACCTACTAAAGCAG		

Smr_Fw	ATAAGTACTGAAGTTATTGGAAGT	285	[18]
Smr_Rv	TTCCGAAAATGTTTAACGAAACTA		

NorA_Fw	TTCACCAAGCCATCAAAAAG	620	[32]
NorA_Rv	CTTGCCTTTCTCCAGCAATA		[13]

NorA_Fw	TTCACCAAGCCATCAAAAAG	95	[32]
NorA_RT(Rv)	CCATAAATCCACCAATCCC		This study

NorB_Fw	AGCGCGTTGTCTATCTTTCC	213	[13]
NorB_Rv	GCAGGTGGTCTTGCTGATAA		

NorC_Fw	AATGGGTTCTAAGCGACCAA	216	[13]
NorC_Rv	ATACCTGAAGCAACGCCAAC		

MepA_Fw	ATGTTGCTGCTGCTCTGTTC	718	[13]
MepA_Rv	TCAACTGTCAAACGATCACG		

MepA_RT(Fw)	TGCTGCTGCTCTGTTCTTTA	198	[13]
MepA_RT(Rv)	GCGAAGTTTCCATAATGTGC		

MdeA_Fw	AACGCGATACCAACCATTC	677	[13]
MdeA_Rv	TTAGCACCAGCTATTGGACCT		

MdeA_RT(Fw)	GTTTATGCGATTCGAATGGTTGGT	155	[33]
MdeA_RT(Rv)	AATTAATGCAGCTGTTCCGATAGA		

16S_27f	AGAGTTTGATCMTGGCTCAG	492	[34]
16S_519r	GWATTACCGCGGCKGCTG		

GrlA_Fw	TGCCAGATGTTCGTGATGGT	339	[35]
GrlA_Rv	TGGAATGAAAGAAACTGTCTC		

GyrA_Fw	TCGTGCATTGCCAGATGTTCG	394	[35]
GyrA_Rv	TCGAGCAGGTAAGACTGACGG		

**^a^**The primers used in the RT-qPCR experiments are indicated by the RT label. Fw: forward; Rv: reverse. For *norB*, *norC *and *smr*, the same set of primers was used for both PCR and RT-qPCR, as well as the primer NorA_Fw.

### PCR amplification of efflux pump genes

DNA fragments internal to five chromosomal and two plasmid encoded efflux pump genes were separately amplified by PCR, using the primers described in Table 3. Reaction mixtures were prepared as described above. Amplification conditions were as follows: DNA was denatured at 94°C for 4 minutes, followed by 35 cycles of denaturation at 94°C for 30 seconds, annealing at 45°C (*norA*) or 53°C (*norB*, *norC*, *mdeA*, *mepA*) for 30 seconds and extension at 72°C for 1 minute, followed by a step of final extension at 72°C for 5 minutes. The PCR reactions for genes *qacA/B *and *smr *were conducted under the following conditions: DNA was denatured at 95°C for 1 minute, followed by 30 cycles of denaturation at 95°C each for 1 minute, annealing at 40°C (*qacA/B*) or 48°C (*smr*) for 1 minute and extension at 72°C for 1 minute, followed by a step of final extension at 72°C for 5 minutes. The amplification products were visualized by 1% agarose gel electrophoresis.

### RNA extraction

For RNA extraction, strains were cultured in TSB media containing ciprofloxacin or EtBr, at ½ their MIC for each strain or in drug-free TSB, and grown until an OD_600 nm _of 0.6. Total RNA was extracted with the RNeasy Mini Kit (QIAGEN), following the manufacturer's instructions. Before extraction of total RNA, cultures were treated with the RNAprotect bacterial reagent (QIAGEN). Contaminating DNA was removed with RNase-free DNase (QIAGEN) by a two hours on-column digestion at room temperature.

### RT-qPCR protocol

Quantitative RT-PCR (RT-qPCR) was performed using the QuantiTect SYBR Green RT-PCR Kit (QIAGEN). The primers used in these assays are described in Table 3. The relative quantity of mRNA corresponding to genes *norA*, *norB*, *norC*, *mepA, mdeA *and *smr *was determined by the comparative threshold cycle (*C_T_*) method [31] in a Rotor-Gene 3000™ thermocycler with real-time analysis software. Relative expression of the efflux pump genes was assessed by two approaches: (i) comparison of the relative quantity of the respective mRNA in the *S. aureus *isolates to the one present in a reference strain, ATCC25923; (ii) comparison of the relative quantity of the respective mRNA in the presence of ciprofloxacin or EtBr (at ½ the MIC) to the drug-free condition. For each strain, three assays were conducted, corresponding to three independent total RNA extractions. Negative controls and genomic DNA contamination controls were included. 16S rDNA was used as reference. Genes showing increased expression of at least four-fold, when compared to the drug-free condition, were considered to be overexpressed [10].

## Competing interests

The authors declare that they have no competing interests.

## Authors' contributions

SSC: helped in the design and performed part of the experiments and wrote the manuscript; CF: performed part of the experiments and participated in the writing of the manuscript; MV: designed the experiments and revised the manuscript; DM: participated in part of the experiments and revised the manuscript; MM: helped in the design of part of the experiments and revised the manuscript; JMC: provided the *S. aureus *clinical isolates and revised the manuscript; LA: helped in the design of part of the experiments and revised the manuscript and IC: designed all the experiments and wrote the manuscript. All authors have read and approved the final manuscript.
