# Correction: Exploring the contribution of efflux on the resistance to fluoroquinolones in clinical isolates of *Staphylococcus aureus*

**DOI:** 10.1186/1471-2180-13-127

**Published:** 2013-06-06

**Authors:** Sofia Santos Costa, Celeste Falcão, Miguel Viveiros, Diana Machado, Marta Martins, José Melo-Cristino, Leonard Amaral, Isabel Couto

**Affiliations:** 1 Grupo de Micobactérias, Unidade de Microbiologia Médica, Instituto de Higiene e Medicina Tropical, Universidade Nova de Lisboa (IHMT, UNL), Rua da Junqueira, 100, 1349-008, Lisbon, Portugal; 2 Centro de Recursos Microbiológicos (CREM), Faculdade de Ciências e Tecnologia, Universidade Nova de Lisboa, Quinta da Torre, 2829-516, Caparica, Portugal; 3 COST ACTION BM0701 (ATENS; 4 Unidade de Parasitologia e Microbiologia Médica (UPMM), Instituto de Higiene e Medicina Tropical, Universidade Nova de Lisboa, Rua da Junqueira, 100, 1349-008, Lisbon, Portugal; 5 UCD School of Public Health, Physiotherapy and Population Science, UCD Centre for Food Safety, Veterinary Sciences Centre, University College Dublin, Belfield Dublin 4, Ireland; 6 Centro Hospitalar Lisboa Norte E.P.E., Instituto de Microbiologia, Instituto de Medicina Molecular, Faculdade de Medicina, Universidade de Lisboa, Avenida Professor Egas Moniz, 1649-028, Lisbon, Portugal

## 

After the publication of our study [1], we became aware that the mutations in the quinolone resistance-determining region (QRDR) of the gene *grlA* were incorrectly described for some of the *Staphylococcus aureus* clinical isolates studied in this work. In particular, isolates SM1, SM10, SM14, SM17, SM25, SM27, SM43, SM46, SM47 and SM48 carry the GrlA double mutation S80Y/E84G; isolate SM52 carries the GrlA mutation S80Y; isolates SM3 and SM5 carry the GrlA double mutation S80F/E84G. The correct data can be found in Table 1.

**Table 1:** **Genotypic and phenotypic characterization of *S. aureus *clinical isolates**

		**QRDR mutations**^**b**^		**MIC (mg/L)**^**c**^											
				**EtBr**			**CIP**			**NOR**			**NAL**		
**Isolate**^**a**^	**PFGE pattern**	**GrlA**	**GyrA**	**No**	**+**	**+**	**No**	**+**	**+**	**No**	**+**	**+**	**No**	**+**	**+**
				**EI**	**TZ**	**CPZ**	**EI**	**TZ**	**CPZ**	**EI**	**TZ**	**CPZ**	**EI**	**TZ**	**CPZ**
ATCC25923	-	WT	WT	6.25	**0.75**	**0.75**	0.25	0.125	0.125	0.5	**0.125**	**0.125**	64	n.d.	n.d.
ATCC25923_EtBr_	-	WT	WT	200	**25**	**12.5**	1	**0.25**	**0.25**	2	**0.25**	**0.25**	64	n.d.	n.d.
**SM1**	A2	S80Y/E84G	S84L	16	**4**	**4**	128	**32**	64	512	**128**	256	256	**64**	**64**
**SM10**	A4	S80Y/E84G	S84L	16	**2**	**4**	128	64	64	512	**128**	**128**	128	64	64
**SM14**	A3	S80Y/E84G	S84L	16	**4**	**4**	256	**32**	128	1024	**128**	**256**	256	**64**	**64**
**SM17**	A4	S80Y/E84G	S84L	16	**4**	**4**	256	**64**	**64**	1024	**256**	512	256	**64**	**64**
**SM25**	A1	S80Y/E84G	S84L	8	**2**	4	128	**32**	64	512	**64**	**128**	256	**32**	**64**
**SM27**	A4	S80Y/E84G	S84L	16	**4**	**4**	256	**32**	**64**	512	**128**	256	256	**64**	**64**
**SM43**	A1	S80Y/E84G	S84L	16	**2**	**4**	128	64	64	512	**128**	**128**	512	256	**64**
**SM46**	A1	S80Y/E84G	S84L	16	**4**	**4**	128	64	64	512	**128**	256	128	64	64
**SM47**	A1	S80Y/E84G	S84L	8	**2**	4	256	**32**	**64**	512	**128**	256	256	**64**	**64**
**SM48**	A1	S80Y/E84G	S84L	8	4	4	256	**32**	**64**	512	**128**	256	256	**64**	**64**
**SM50**	B1	S80F/E84K	S84L	8	**1**	**2**	64	**16**	**16**	256	**32**	**64**	128	64	64
**SM52**	C1	S80Y	S84L	16	**1**	**2**	16	8	8	64	32	32	128	**32**	64
SM2	B2	S80F/E84K	S84L	8	**2**	**2**	32	16	16	128	**32**	**32**	64	**16**	64
SM3	E1	S80F/E84G	S84L	1	1	1	16	8	8	64	32	32	64	**16**	**16**
SM4	E2	S80F	S84L	4	2	**1**	8	8	8	64	32	32	64	32	64
SM5	E3	S80F/E84G	S84L	4	2	**1**	32	16	16	128	64	64	64	32	32
SM6	A5	S80F	E88K	4	2	**1**	16	16	16	64	32	32	64	32	32
SM7	E1	S80F	S84L	2	2	1	8	8	4	64	32	32	128	**32**	64
SM8	A5	S80F	E88K	4	2	**1**	16	8	16	128	64	64	128	**32**	64
SM12	E1	S80F	S84L	2	2	1	16	8	8	64	32	32	128	**32**	64
SM16	A6	S80F	E88K	4	2	**1**	16	16	16	128	**32**	64	64	32	64
SM22	A1	S80Y/E84G	S84L	8	4	4	128	**16**	**32**	512	**128**	**128**	64	32	64
SM34	D1	S80F/E84K	S84L	4	2	2	64	**16**	32	64	**16**	32	32	16	32
SM36	E1	S80F	S84L	4	2	2	16	8	8	64	**16**	32	128	**32**	64
SM40	E1	S80F	S84L	8	4	4	32	32	32	512	**128**	**128**	16	8	16

^a^Isolates in bold correspond to the EtBrCW-positive isolates. ^b^WT: wild-type; S: serine; F: phenylalanine; E: glutamate; K: lysine; Y: tyrosine; L: leucine; G: glycine. ^c^Values in bold-type correspond to a MIC decrease of ≥ four-fold in the presence of the efflux inhibitor (EI) in comparison to the values with no EI [10]. The concentration of each EI used is defined in the Methods section. EtBr: ethidium bromide; CIP: ciprofloxacin; NOR: norfloxacin; NAL: nalidixic acid; TZ: thioridazine; CPZ: chlorpromazine; n.d.: not determined.

All clinical isolates included in this study were selected upon a ciprofloxacin resistance phenotype and all the 25 representative isolates screened for mutations conferring fluoroquinolone resistance carried QRDR mutations in both *grlA* and *gyrA* genes. All the mutations found have been described in literature as associated with fluoroquinolone resistance in *S. aureus* clinical isolates [2]. As stated previously in our study, the majority of the isolates presented a double mutation in GrlA together with a single mutation in GyrA. Eleven isolates carried the GrlA and GyrA mutations S80Y/E84G and S84L, respectively; three isolates carried mutations GrlA S80F/E84K and GyrA S84L and two isolates carried mutations GrlA S80F/E84G and GyrA S84L.The remaining nine isolates carried a single mutation in both genes, in three distinct arrangements (Table 1).

Despite this correction in the QRDR mutations carried by some of the isolates studied, the main findings of our study are not altered. In particular, our data show the potential role played by efflux systems in the development of resistance to fluoroquinolones in clinical isolates of *S. aureus*, independently of the mutations occurring in the target genes.

We apologize for any inconvenience that this may have caused to the readers.
